# Synthesis, Characterization, and Three-Dimensional Structure Generation of Zinc Oxide-Based Nanomedicine for Biomedical Applications

**DOI:** 10.3390/pharmaceutics11110575

**Published:** 2019-11-04

**Authors:** Su-Eon Jin, Hyo-Eon Jin

**Affiliations:** 1 College of Pharmacy, Yonsei University, Incheon 21983, Korea; hibis1@gmail.com; 2 College of Pharmacy, Ajou University, Suwon 16499, Korea

**Keywords:** zinc oxide nanoparticles, synthesis, physicochemical characteristics, three-dimensional structure, biomedical application

## Abstract

Zinc oxide (ZnO) nanoparticles have been studied as metal-based drugs that may be used for biomedical applications due to the fact of their biocompatibility. Their physicochemical properties, which depend on synthesis techniques involving physical, chemical, biological, and microfluidic reactor methods affect biological activity in vitro and in vivo. Advanced tool-based physicochemical characterization is required to identify the biological and toxicological effects of ZnO nanoparticles. These nanoparticles have variable morphologies and can be molded into three-dimensional structures to enhance their performance. Zinc oxide nanoparticles have shown therapeutic activity against cancer, diabetes, microbial infection, and inflammation. They have also shown the potential to aid in wound healing and can be used for imaging tools and sensors. In this review, we discuss the synthesis techniques, physicochemical characteristics, evaluation tools, techniques used to generate three-dimensional structures, and the various biomedical applications of ZnO nanoparticles.

## 1. Introduction

Metal-based drugs, generally including inorganic nanomaterials, have been studied as a next-generation nanomedicine [1,2]. Some of these drugs include iron oxide (Fe_3_O_4_ or γ-Fe_2_O_3_), titanium dioxide (TiO_2_), cerium dioxide (CeO_2_), copper oxide (Cu_2_O or CuO), silica (SiO_2_), gold, silver, platinum, and zinc oxide (ZnO) nanoparticles (NPs) [1,2,3,4]. These NPs demonstrate their unique pharmaceutical characteristics and novel pharmacological functions [5,6,7]. Metal-based nanotherapeutics with controllable features such as particle size and porosity, are valuable for biomedical applications of drug delivery and therapeutic activity.

Zinc oxide NPs have been highlighted as promising metal-based nanodrugs due to the fact of their biocompatibility, selectivity, and high potency [2,6,7]. They have a wide band gap energy (3.3 eV) and a high excitation binding energy (60 meV) at room temperature with thermal and mechanical stability [8,9]. Zinc oxide NPs have been extensively used in applications related to optical, chemical sensing, semiconducting, and piezoelectric research [10,11]. They also have photocatalytic functions that allow them to be used in purification and disinfection processes [12,13,14]. Although ZnO is categorized as “generally recognized as safe (GRAS)” by the US Food and Drug Administration [15], ZnO NPs still have toxicity issues [16].

Despite the toxicity, ZnO NPs have been conventionally used in pharmaceuticals, cosmetics, and medical devices of adhesives, mastics, and sealants [17,18,19] (Table 1). In the pharmaceutical industry, ZnO NPs are used in soaps, toothpaste, ointments, dental inlays, and powders [20]. Zinc oxide NPs are also used in hair and skin powders, creams, ultraviolet (UV) radiation-blocking sunscreen lotions, and burn ointments [20,21]. Surgical and industrial adhesives containing ZnO NPs (e.g., Neoprene adhesive [22]) are used for medical devices.

**Figure 1:**
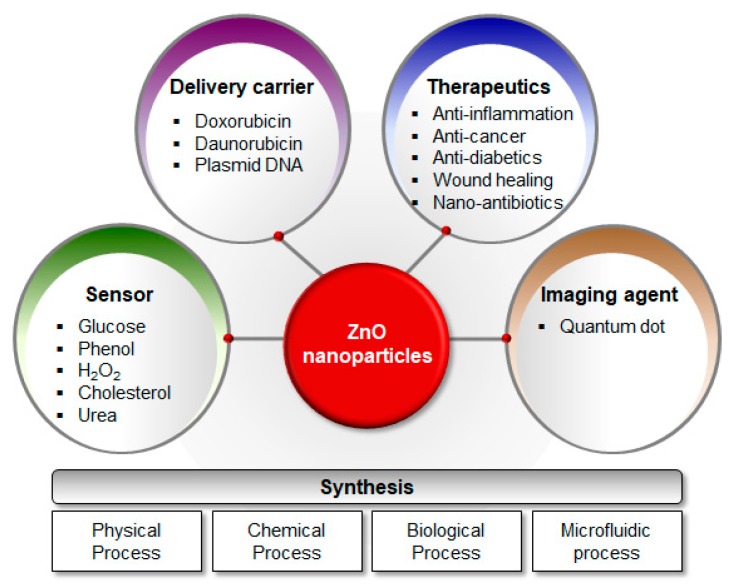
Schematic diagram of synthesis techniques and applications for ZnO NPs. Zinc oxide NPs are synthesized via (i) physical, (ii) chemical, (iii) biological, and (iv) microfluidic processes. They are extensively applied as (i) delivery carriers, (ii) therapeutics, (iii) sensors, and (iv) imaging agents. Abbreviations: ZnO, zinc oxide; NPs, nanoparticles.

Zinc oxide NPs have also been investigated as drug delivery carriers, therapeutics, and diagnostics for human biomedical applications due to the fact of their biocompatibility [23,24,25,26,27] (Figure 1). Zamani et al. [25] reported mesoporous ZnO–graphene oxide (GO) combined with TiO_2_ NPs (TiO_2_@ZnO–GO NPs) for the targeted drug delivery to the colon. The TiO_2_@ZnO–GO NPs showed a pH-dependent drug release, in which the rate of release was higher at a neutral pH than at an acidic pH. Doxorubicin, daunorubicin, and plasmid DNA were conjugated to ZnO NPs to improve targeted delivery of cancer therapeutics [28,29,30]. Zinc oxide NPs have also been developed as therapeutics for the treatment of bacterial infections, diabetes, wounds, and inflammation [24,31]. In terms of diagnostic applications, ZnO nanostructures have been studied as biosensors, including nanowires for glucose detection [32], and core–shell nanorods for detecting UV radiation and hydrogen [33]. 

The unique morphology and structure of ZnO NPs and their networks are generated depending on synthesis techniques [34,35]. The following one-dimensional ZnO structures have been reported: nanocombs [36,37], nanorods [38,39], nanobelts [40,41], nanoneedles [42,43], and nanowires [44,45]. Nanoplates/nanosheets [46,47] and nanopellets [48] are two-dimensional structures, while nanoflowers [49] and snowflakes [50] are three-dimensional structures described in the literature. Moreover, ZnO NPs and aggregates can be built into three-dimensional network structures with hierarchical porosity [13,51]. 

In this review, we summarized the synthesis techniques, physicochemical properties (including the tools used to evaluate these properties), and unique structures of ZnO NPs. We categorized the synthesis techniques of ZnO NPs into the following categories: conventional (e.g., physical, chemical, and biological methods) and microfluidic reactor-based methods. In the discussion regarding the physicochemical properties of ZnO NPs, we describe representative evaluation tools of X-ray diffraction (XRD), scanning electron microscopy (SEM), transmission electron microscopy (TEM), and Brunauer–Emmett-Teller (BET) analysis. We also explore the three-dimensional ZnO NP structures and their fabrication methods including conventional synthesis techniques, biotemplating, and self-assembly. We focused particularly on the multilevel porous three-dimensional structures that are used for biomedical applications. We further discussed the current biomedical applications of ZnO NPs.

## 2. Synthesis Techniques for ZnO NPs

Zinc oxide NPs can be synthesized using either conventional or non-conventional methods [52,53]. While physical, chemical, and biological (green) synthesis techniques are included among the conventional methods, microfluidic reactor-based synthesis is introduced as a non-conventional method. The representative advantages and disadvantages of these synthesis techniques for ZnO NPs are listed in Table 2.

### 2.1. Conventional Methods

Both top-down and bottom-up approaches can be used to synthesize nanomaterials [9,54,55]. The top-down approach involves physically slicing or cutting bulk materials into nano-sized materials [54]. The bottom-up approach, on the other hand, uses atoms and molecules to build nanostructures through chemical or biological synthesis, or controlled deposition and growth [55]. Biological synthesis, which is otherwise referred to as “green synthesis,” is desirable due to the simplest, most efficient, reproducible, and ecologically responsible option. However, the mechanism of green synthesis is not yet fully understood [56].

#### 2.1.1. Physical Methods

Physical methods include arc plasma, thermal evaporation, physical vapor deposition, ultrasonic irradiation, and laser ablation. These processes are chemically pure and technically simple, which makes them ideal for carrying out industrial processes at high production rates [57,58,59,60]. Arc plasma, which is based on electrical arc discharge synthesis, is one of the most commonly used physical methods for converting bulk materials into nanomaterials via condensation and evaporation [61]. Peng et al. [62] described a plasma method that involved reacting Zn powder with oxygen gas at 0.5–50 L/min to produce wool-like ZnO nanorods. Using thermal evaporation, ZnO thin films [63] and nano/micro ZnO rods [64] were synthesized via deposition on substrates. Fouad et al. [63] reported the synthesis of highly oriented needle-like ZnO crystals (thickness: 10–80 nm) that showed the photocatalytic decomposition of azo-reactive dye at a deposition and oxygen temperature of 350–650 °C for 10 to 30 min. Zhang et al. [64] also reported the synthesis of nano/micro ZnO rods using simple thermal evaporation at 650–850 °C for 60 to 120 min. Zinc oxide nanowires on Al_2_O_3_ substrate that were fabricated via physical vapor deposition between 450 and 600 °C of low growth temperature presented with a high-quality structure and crystallinity [65]. Using ultrasonic irradiation for 75 to 270 min, Yadav et al. [66] sonochemically synthesized histidine (capping agent)-based ZnO NPs with a tunable band gap. Thareja et al. [67] used pulsed a laser ablation technique with an Nd:YAG laser (10 Hz, 130 mJ/pulse, 5 ns/pulse duration) to produce a colloidal suspension of ZnO NPs. 

#### 2.1.2. Chemical Methods

Chemical methods include microemulsion, sol–gel, precipitation, hydrothermal, solvothermal, and chemical vapor deposition [9,53]. Wet chemical synthesis, which is based on the physical states of the solid and liquid phases, is the most commonly used method for producing NPs [9]. During industrial-scale wet chemical synthesis, capping agents/stabilizers are used extensively in spite of their toxicity to control particle size and to prevent the agglomeration. Triethylamine (TEA), oleic acid, thioglycerol, and polyethylene glycol are representative capping agents/stabilizers although they have immunogenic and apoptotic/necrotic potential [68]. In a microemulsion, stabilizers are used to generate thermodynamically stable fluid droplets from immiscible phases of hydrocarbon and water. Fricke et al. [69] reported a method for mini-emulsion-based ZnO NP synthesis using TEA to control the size (<200 nm) and shape (hexagonal wurtzite crystal) of ZnO NPs. Using sol–gel synthesis, Valdez et al. [70] created dodecylamine (DDA)-capped ZnO nanocrystals with a low surface density of DDA (25%) due to the hydroxide groups (protons) on the surface of the ZnO NPs. This precipitation technique involves a reaction initiated using a source of zinc and alkali (sodium hydroxide, potassium hydroxide, ammonium, or urea) to promote aggregation. The precipitates of ZnO NPs are then collected by filtration or centrifugation. Oliveira et al. [71] described the controlled precipitation of ZnO NPs from zinc nitrate and zinc sulfate with sodium hydroxide. Demir et al. [72] reported the precipitation of ZnO nanocrystals using the acid-catalyzed esterification of zinc acetate in a mixture of l-pentanol and m-xylene. Hydrothermal and solvothermal techniques involve the material synthesis under heated aqueous and non-aqueous conditions, respectively [73]. The parameters of the hydrothermal and solvothermal techniques used for the synthesis affect the structure, morphology, composition, and assembly of the resulting ZnO NPs. Aneesh et al. [74] synthesized green photoluminescent ZnO NPs using the hydrothermal technique. Santos et al. [75] described the solvothermal technique to synthesize gallium–indium-ZnO NPs for electrolyte-gated transistors. Chemical vapor deposition, which is a simple and effective method, has also been used for ZnO NP synthesis. However, this method has been known to produce heterogeneous growth. Noothongkaew et al. [76] reported the synthesis of green photoluminescent ZnO nanowalls on a silicon (Si) substrate.

#### 2.1.3. Biological Methods

Biological methods are promising alternatives to physical and chemical synthesis methods because they are eco-friendly [77]. Microorganisms (bacteria, fungi, yeast, algae, and phage), DNA, proteins, and plant extracts have been studied extensively for the biological synthesis of ZnO NPs [78,79]. However, the mechanisms of producing ZnO NPs via biological synthesis are not fully understood yet. 

Zinc oxide NPs can be synthesized in appropriate microorganisms using various enzymes and biochemical pathways. Bacteria including *Bacillus megaterium* NCIM2326 [33], *Halomonas elongata* IBRC-M 10214 [80], *Sphingobacterium thalpophilum* [81], and *Staphylococcus aureus* [82] have been used to synthesize ZnO NPs (10–95 nm; rod/cubic, multiform, triangle, acicular) for antimicrobial agents. Fungal species including *Aspergillus niger* [83] and *Candida albicans* [84], can also synthesize ZnO NPs. Zinc oxide NPs synthesized from fungi had spherical to quasi-spherical shapes of 61 nm and 25 nm, respectively. These NPs were used for antimicrobial applications and steroidal pyrazoline synthesis. *Pichia kudriavzevii* [85] and *Pichia fermentans* JA2 [86] as yeast systems can also synthesize ZnO NPs. In yeast, hexagonal wurtzite and smooth/elongated ZnO NPs (10–61 nm) were produced and these NPs were used for antimicrobial applications. In algae, *Chlamydomonas reinhardtii* [87] and *Sargassum muticum* [88] were used to synthesize ZnO NPs. These algal species produced nanorods/nanoflowers (55–80 nm from HR-SEM; 21 nm from XRD) and hexagonal wurtzite NPs (30–57 nm from FE-SEM; 42 nm from XRD). A phage-directed system of M13 bacteriophage exposing ZnO-binding peptides on pIII or pVIII phage coat protein produced photoluminescent wurtzite ZnO NPs [89].

The DNA, amino acids, and proteins can also be used for the ZnO NP synthesis [9,52]. Li et al. [90] used DNA to guide the synthesis of ZnO NP chains and to control their growth. L-alanine [91], gelatin [92], and egg albumin [93,94] were used for ZnO NP synthesis. Gharagozlou et al. [91] described the L-alanine-assisted synthesis of ZnO NPs between 50–100 nm in size (TEM; SEM). Gelatin was also used to synthesize ZnO NPs (Zn, 59.10%; O, 28.93%) of 20 nm in size; these NPs showed high antibacterial and anti-angiogenic activities [92]. Ambika et al. [93] described the use of egg albumin to synthesize ZnO NPs that were spherical and hexagonal wurtzite. These albumin-based NPs were measured at 16 nm (XRD), 10–20 nm (TEM), and 8–22 nm (AFM). Other reports documenting egg albumin-capped ZnO NPs described them as spherical, hexagonal wurtzite nanocrystals with a hydrodynamic diameter of 34.2 nm [94]. 

Plant extracts are attractive for use in the biological synthesis of metal oxide NPs due to the presence of components such as flavonoids, terpenoids, and polysaccharides [9]. *Calotropis procera* leaf extract [95], *Matricaria chamomilla* (flower)/*Olea europaea L*. (leaf)/*Lycopersiconesculentum M.* (fruit) extract [96], *Pelargonium graveolens* leaf-extracted geranium oil [97], and *Thymus vulgaris* leaf extract have been used to synthesize ZnO NPs and ZnO-Ag nanocomposites. Zinc oxide NPs synthesized from plant extracts have been applied for dye photodegradation, antimicrobial applications, and solar photocatalysis.

### 2.2. Non-Conventional Method: Microfluidic Reactor-Based Synthesis

A microfluidic reactor is a miniaturized, non-conventional synthesis tool which may be used for bench-top material fabrication [73,98]. The mechanisms of actions, critical parameters, advantages, and particular cases are described in microfluidic ZnO NP synthesis. Firstly, microfluidic reactor systems manipulate and control the flow in reaction environments, thus allowing for better control of the reaction [98]. Such a system usually attaches to a lab-on-a-chip or micro-total-analysis system. Since the microfluidic reactor mixes reactants on a microscale, viscosity is the major factor affecting flow rather than inertial forces (low Reynolds number < 10^2^) [99,100]. Thus, in the microfluidic environment, mixing occurs through diffusion and laminar flow. 

The reaction temperature should also be tightly controlled in a microfluidic reactor when synthesizing NPs [73,100]. In the microchannel of a microfluidic reactor, the reactions are controlled with reducing agents and metal salts at low temperatures (15–20 °C). Dynamic precursors are formed via reduction after the reactants are mixed. Finally, NP nucleation and growth occur at a higher temperature (80–90 °C). To produce high-quality NPs with a high degree of crystallinity and narrow size distribution, each step of nanoparticle generation should be controlled within a narrow time frame and terminated at the desired stage.

Microfluidic reactor systems have several advantages in NP synthesis [101]. Compared with classical and macroscale synthesis, a microfluidic reactor uses small reagent volumes and offers selectivity, environmental friendliness, short reaction time, a small footprint, and improved safety [102]. These systems have been used in academia and industry for reaction optimization [101,102]. Specifically, controlling the flow (e.g., continuous or segmented flows) alters the reaction conditions (e.g., temperature, time, and reagent concentrations) in the microfluidic reactor to produce high-quality products with improved characteristics and enhanced performance [103,104]. Metal oxide NPs, semiconductors, and quantum dots (QDs) are typical products of microfluidic reactor-based synthesis.

Microfluidic reactor systems have also been used to synthesize ZnO NPs. In a microfluidic reactor, ZnO NPs/nanowires were synthesized using a hydrothermal method [103,105,106,107]. Azzouz et al. [103] reported the synthesis of ZnO nanowires from ZnO seeds and explored their ability to remove volatile organic compounds from water. Joo et al. [105] also reported the bottom-up device fabrication for producing ZnO nanowires in a continuous flow from ZnO seeds. Kraus et al. [106] used a segmented flow for the synthesis of ZnO NPs. They generated microfluidic segments of droplet-like small reaction mixture portions at high flow rates. Using a static micromixer, they enhanced internal convection by promoting heat exchange between the reaction mixture and channel environment. On the other hand, Zukas and Gupta [107] used a two-phase co-flow system in a droplet flow reactor with a T-junction for the synthesis of ZnO NPs.

## 3. Physicochemical Characterization and Tools

The Organization for Economic Co-operation and Development (OECD) recommends that engineered nanomaterials undergo a physicochemical property evaluation as a pre-requisite for toxicological assessment [108,109]. The OECD recommends investigating the following physicochemical properties: agglomeration/aggregation, catalytic potential, composition, concentration, crystalline phase, dustiness, fat solubility/oleophilicity, grain size, hydrodynamic size/particle size/size distribution, length, purity, shape, specific surface area, surface chemistry, water solubility/hydrophilicity, and zeta potential [108]. Table 3 summarizes the physicochemical characteristics of ZnO NPs and the analysis tools. The physicochemical results for engineered nanomaterials are needed to predict toxicological risks in vitro and in vivo [109]. The physicochemical properties of ZnO NPs and their representative evaluation tools are also described below.

### 3.1. Appearance, Crystallinity, Particle Size, Morphology, and Porosity

Zinc oxide NPs (81.38 g/mol, m.p. 1975 °C) are a white, colorless, and odorless solid. Zinc oxide crystal structures mainly take after hexagonal wurtzite and cubic zinc blended forms [9,110]. The hexagonal wurtzite form in which each tetrahedral Zn atom is surrounded by four oxygen atoms or vice versa is common and generally stable [35,110]. Zinc oxide NPs are less than 200 nm in diameter and are used in cosmetics, foot care products, whitening agents, and ointments [68,110]. As previously mentioned, ZnO NPs have one-, two-, or three-dimensional structures. They also generate aggregates and can self-assemble into three-dimensional networks with multilevel porosity [13,68,111].

### 3.2. Characterization Tools

Characterization tools are necessary to identify the properties of engineered nanomaterials. Some tools used to determine the crystallinity, morphology, particle size/size distribution, and surface characteristics (specific surface area and porosity) of ZnO NPs include XRD, SEM, TEM, and BET analysis.

#### 3.2.1. X-ray Diffraction (XRD)

X-ray diffraction is a well-established technique for analyzing the size, shape, and crystal structures of inorganic, carbon-based, or complex crystalline materials [108,109]. It offers high spatial resolution at the atomic scale, but it is limited to crystalline materials and has a lower intensity compared to electron diffraction. For ZnO NPs, a pure hexagonal wurtzite structure was identified using diffraction peaks (2θ degree) and attributed to the following Miller–Bravais indices: (100), (002), (101), (102), (110), (103), (200), (112), and (201) (JCPDS No.89-0510 or JCPDS No.36-1541) [112,113,114]. Bindu and Thomas [112] analyzed the lattice strain in ZnO NPs with crystalline sizes of 27.49 nm, 35.35 nm, 36.28 nm, 36.09 nm, and 34.55 nm as calculated by Scherrer method, the uniform deformation model, uniform stress deformation model, and uniform deformation energy density model of the Williamson–Hall method, and a size-strain plot. The crystal size of those ZnO NPs was measured at 30 nm using TEM. Khalafi et al. [113] synthesized pure ZnO NPs using a *Chlorella* aqueous extract and reported a hexagonal wurtzite structure (19.44 nm; calculated from Debye–Scherrer equation) as determined by the XRD pattern. Abdullayeva et al. [115] investigated the crystallinity of nanoflower-, nanosheet-, and nanorod-like three-dimensional ZnO nanostructures. According to the XRD patterns, all ZnO nanostructures were the hexagonal wurtzite type from the (100), (002), and (101) of Miller–Bravais indices.

#### 3.2.2. Scanning Electron Microscopy (SEM)

Scanning electron microscopy is a high-resolution method for estimating size, size distribution, shape, aggregation, dispersion (cryo-SEM), and crystallinity (electron backscattering detection) [108,109]. It may be used to analyze inorganic, organic, carbon-based, biological, and complex materials and to determine whether they are spherical or equiaxial particles, tubes, flakes, rods, fibers, or of any other shape. Scanning electron microscopy is limited to the analysis of conductive or coated materials under non-physiological conditions. The cryogenic method is required for biomaterials. Various ZnO NP shapes have been reported from SEM analyses, including spheres and rods [13,32,116,117]. Sphere-type ZnO NPs less than 50 nm in diameter have also been reported [116]. Other spherical ZnO NPs produced an aggregate network on a Si wafer using a layer-by-layer structure [13,111]. Zinc oxide NPs that were used in electrochemical biosensors for detecting glucose, were shaped as nanocombs, nanorods, nanofibers, nanowires, and nano-nails [117].

#### 3.2.3. Transmission Electron Microscopy (TEM)

Transmission electron microscopy measures size and size distribution and confirms the nanomaterial shapes with higher resolution compared to SEM [108,109]. Aggregation, dispersion (environmental TEM), and crystal structure can also be determined by TEM. The TEM technique is limited to very thin samples under non-physiological conditions. It can be used to visualize inorganic, organic, carbon-based, biological, and complex materials as spherical and equiaxial particles, tubes, flakes, rods, or fibers. The size, size distribution, crystalline structures, and aggregates of ZnO NPs have been analyzed using TEM [46,118,119]. The TEM technique is extensively used to determine the size, size distribution, and morphology of ZnO NPs based on the stabilizer (glycerol)-to-zinc source ratios during the synthesis [46,109]. Li et al. [118] reported the layer-by-layer growth of ZnO nanopillar crystals using in situ, high-resolution TEM. Ludi and Niederberger [119] also used TEM to demonstrate the nucleation and growth of ZnO NPs, including the hexagonal pyramid and oleic acid-stabilized, cone-shaped ZnO nanocrystals in liquid media. 

#### 3.2.4. Brunauer–Emmett-Teller (BET) Analysis

Brunauer–Emmett-Teller analysis provides the specific surface area and porosity of spherical and equiaxial particles of inorganic, carbon-based, and complex materials [120]. This technique is limited to the analysis of volatile compound-free materials. Furthermore, BET cannot distinguish between particles and nonparticulate porous materials. Mesoporous ZnO thin films were found to have a specific surface area of 14–140 m^2^/g depending on the synthesis techniques [121]. Zafar et al. [122] reported spherical ZnO NPs with a specific surface area of 49.36 m^2^/g that could be used for the removal of adsorptive azo dyes, such as methyl orange and amaranth. Lu et al. [123] described three-dimensional macroporous network structures of ZnO that were synthesized as dried gels. These structures had specific surface areas of 131 m^2^/g, 50 m^2^/g, 20 m^2^/g, and 18 m^2^/g, before and after heat treatment at 320 °C, 360 °C, and 400 °C, respectively.

## 4. Three-Dimensional Structure Generation by Nanofabrication

### 4.1. Three-Dimensional Network Structure with Multilevel Porosity

**Figure 2:**
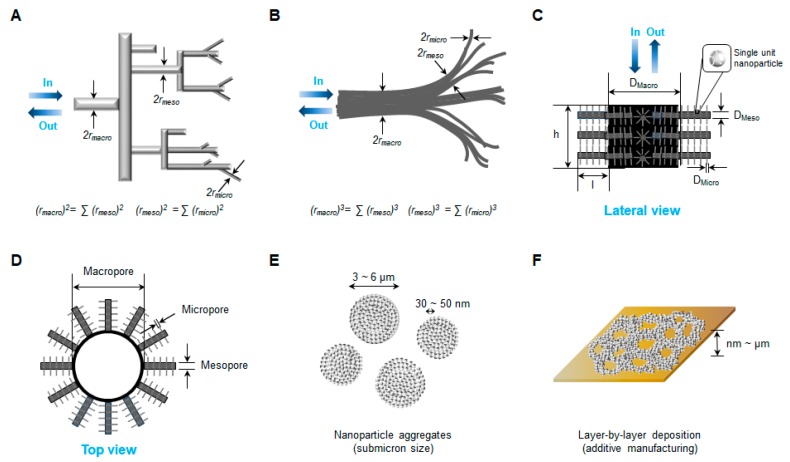
Schematic diagrams of multilevel porosity in ZnO NPs and their self-assembled three-dimensional structures for enhancement of photocatalysis. Multilevel pores in (**A**) insect and (**B**) leaf branching systems, followed by Murray’s law, (**C**) lateral and (**D**) top views of hierarchically porous ZnO NP network model, (**E**) ZnO NP aggregates and (**F**) layer-by-layer structure network of ZnO NPs on a solid plate. Abbreviations: ZnO, zinc oxide; NPs, nanoparticles.

Zinc oxide NPs have been shown to form three-dimensional networks with multilevel porosity [51,111,123]. Pores are defined by the International Union of Pure and Applied Chemistry (IUPAC) in terms of size: micropores (<2 nm), mesopores (2 nm–50 nm), and macropores (>50 nm) [124,125]. A micropore is also known as a “nanopore”. Multilevel or multiscale porosity is usually bimodal (micro-meso, meso-micro or micro-macro) or trimodal (micro-meso-macro or meso-meso-macro) [126]. Hierarchically porous structured materials are highly porous, multiscale, and interconnected with a large surface area and low density. In experimental models, hierarchical pores follow Murray’s law which is used to determine the sizes of vessels in in the architecture of transport systems for insects (Figure 2A) and leaves (Figure 2B) [111,127]. Using a hierarchically porous material model, macro-, meso-, and microporous (nanoporous) channel modalities are designed to meet specific performance goals (lateral view, Figure 2C; top view, Figure 2D). Networks of ZnO NPs are tuned to provide catalysis via light scattering, potential harvesting, multiple internal reflections of NP aggregates (Figure 2E), and layer-by-layer structures (Figure 2F) [128]. Wang et al. [129] reported the gas-sensing activity of nest-like, hierarchically porous ZnO structures. The specific surface area and pore size of the nest-like ZnO structures synthesized by a one-pot hydrothermal method, were 36.4 m^2^/g and 3–40 nm, respectively. Lei et al. [130] also described highly efficient dye (Congo red) adsorption by hierarchically porous ZnO microspheres designed to remove anionic organic dyes from wastewater. The specific surface area of those hierarchically porous ZnO microspheres was 57 m^2^/g, and their maximum adsorption was 334 mg/g of Congo red. Besides gas sensing and photocatalytic degradation, hierarchically porous ZnO NP structures can be applied to drug delivery and tissue engineering [131,132]. Leone et al. [131] reported micro-metric or sub-micrometric aggregates of spherical NPs loaded with ibuprofen for antibacterial drug delivery, which were effective in preventing the growth of *S. aureus* > *C. albican* > *K. pneumoniae*. Pérez et al. [132] used osteostatin-loaded mesoporous bioactive SiO_2_–CaO–P_2_O_5_ glass containing 4–5% ZnO as three-dimensional porous scaffolds for promoting bone regeneration. Osteostatin in ZnO-mesoporous structured glass scaffolds promoted osteogenesis in MC3T3-E1 cells.

### 4.2. Nanofabrication Techniques

#### 4.2.1. Conventional Methods of Nanofabrication

Hierarchically porous materials can be fabricated using a variety of procedures, including dual surfactant templating, colloidal crystal templating, polymer templating, bioinspired processing, emulsion templating, freeze drying, phase separation, breath figures, selective leaching, replication, zeolitization, sol–gel control, and post-treatment [133,134,135,136,137,138,139,140]. Fabrication technologies are divided into four categories: basic (surfactant templating, replication, sol–gel control, and post-treatment), chemical (emulsion templating, phase separation, zeolitization and self-formation), replication-related chemical (colloidal templating, bioinspired processing and polymer templating), and physical–chemical (supercritical fluids, freeze drying, breath figures, and selective leaching) methods [140]. Self-formation in chemical fabrication via a spontaneous phenomenon produces hierarchically porous materials from a metal alkoxide (reactant) and a surfactant (template) in a solvent (water and co-solvent). This method has the advantages of direct production, simplicity, and facile scale-up. Furthermore, this technology is also easy to combine with other fabrication methods.

#### 4.2.2. Non-Conventional Methods of Nanofabrication

##### Biotemplating

Biotemplating uses biological structures or replicates the morphological and functional characteristics of biological species to guide the assembly or array of inorganic nanomaterials [141]. Proteins, biopolymers, natural scaffolds, and microorganisms are used as biotemplates to obtain the required morphology. After the nanomaterials are synthesized, biotemplates should be removed for purification. 

Prakash et al. [142] used albumen as a biotemplate for ZnO NP films designed to sense acetic acid in aqueous mixtures. Gelatin was also reported as a biotemplate to obtain the desired crystal structure using a biomimetic method [143]. It assisted with the hydrothermal synthesis of star-like ZnO NPs by facilitating the self-assembly of nanorods. The resulting product was able to perform photocatalytic degradation of methyl orange when exposed to UV irradiation. Oudihia et al. [144] described a biological method in which *Azadirachta indica* (neem) leaves acted as cellulose biotemplates for capping in solvents during the synthesis of blue-emitting ZnO nanostructures at 12–36 nm in size. Silk fibroin fibers were also used to synthesize biotemplated, photoluminescent ZnO NPs [145].

Among natural scaffolds, eggshell membrane [146], rice [147], and banana stalks [148] have been used as biotemplates for the synthesis of ZnO NPs. Camaratta et al. [146] investigated eggshell membrane-based biomimetization for the synthesis of ZnO nanostructures using zinc acetate, zinc nitrate, and zinc chloride as precursors. Ramimoghadam et al. [147] used uncooked rice as a soft biotemplate for the hydrothermal synthesis of hexagonal wurtzite ZnO NPs with flake-like, small flower-like, tooth-like, and star-like structures. Upneja et al. [148] reported banana stalks as a source of biofuel and biotemplate for the hydrothermal synthesis of ZnO nanostructures with a ~20 m^2^/g specific surface area.

Microorganisms are attractive, cost-effective, and versatile biotemplates for the bottom-up fabrication of biologically inspired heterogenous nano/micro-structures [149]. Bacteria [26,149] and viruses [150] have been used as biotemplates for ZnO synthesis. Microzyme species have been used for the synthesis of hollow ZnO spheres that were applied to detect acetone [26,149]. Stitz et al. [150] reported the use of tobacco mosaic virus (TMV)-based piezoelectric ZnO films for promoting biomineralization and acting as biomimetics. The TMV template had electromechanical properties due to the formation of dipoles in the protein structure, which was a non-centrosymmetric structure of a polar protein in the axial plane of the virus fibers.

##### Nanofabrication via Self-Assembly

Self-assembly involves the formation of an organized structure or pattern based on conventional ionic, covalent, metallic, hydrogen, and coordination bonds. These bonds are built from weaker interactions such as van der Waals and Casimir; π–π and hydrophobic; and colloidal and capillary, magnetic, electrical or optical forces [151,152,153]. This approach is a smart nanofabrication technique based on material properties. Jin et al. [13] described a self-assembled three-dimensional network structure consisting of ZnO NPs and aggregates. This network was prepared by dripping ZnO NP hexane suspensions onto a Si wafer [13,151]. Zheng et al. [111] also reported that ZnO NPs developed a hierarchical, trimodal porosity network on solid Si wafer substrate via self-assembly [152,153] after ZnO NP hexane suspensions were dripped onto the substrate and the hexane was evaporated. Du et al. [154] reported the self-assembly of hexagonal, grid-like ZnO lamellae that were synthesized with ο-phthalic acid using a hydrothermal technique. The grid-like ZnO lamellae were prepared via the interlinked self-assembly of ZnO NPs in ethanol, which were coated onto an alumina ceramic tube containing a gold electrode after air-drying (60 °C, 1 h) and heating in an electric furnace (350 °C, 1.5 h). Zena et al. [155] also reported self-assembled and monolayer-based hydrothermal fabrication of ZnO nanorods on indium tin oxide substrates. Liu et al. [156] described the synthesis of precursor-directed and self-assembled porous ZnO nanosheets. The ZnO nanosheets had a unique parallelogram morphology, which were formed following an alkalization reaction and self-assembly using didodecyldimehtylammonium bromide as a surfactant. These ZnO nanosheets served as a high-performing semiconductor substrate for surface-enhanced Raman scattering. In the case of titania, Han et al. [157] reported an evaporation-induced self-assembly technique that used titanium (IV) tetraethoxide as a precursor, obtaining a hierarchically porous titania surface with macro- and mesopores for cell adhesion, proliferation, and mineralization.

## 5. Biomedical Applications

Zinc oxide NPs have been studied for biomedical applications because they have shown anticancer, antidiabetic, antimicrobial, anti-inflammatory, and wound healing activities. They have also been used in imaging agents and biosensors [24,158] (Table 4). Various three-dimensional structures of ZnO NPs and aggregates affect biomedical activity by modulating the surface characteristics of the hierarchically porous architectures [13,111,126,158]. These hierarchically porous architectures enhance mass transfer via light scattering and multiple reflections caused by micro-/macrochannels in the nanomaterials [159,160].

### 5.1. Anticancer Activity

Zinc is an essential trace element that regulates the activity of many enzymes to maintain homeostasis in the body [1,161]. Zinc also plays a role in humoral and cellular immunity, which protects cells against cancer. Zinc deficiency causes the initiation and propagation of cancer cells via DNA mutation and p53 disruption [1,2,162]. Zinc oxide NPs have enhanced permeability and retention (EPR) effects toward cancer cells compared to bulk zinc materials and can kill cancer cells through the generation of reactive oxygen species (ROS) [1,5,163]. Zinc oxide NPs were investigated as standalone agents against HepG2 (hepatocellular carcinoma), PC3 (prostate cancer), A549 (non-small cell lung carcinoma), B16F10/A375 (melanoma), HeLa (cervix adenocarcinoma), HNSCC (head and neck squamous cell carcinoma), LoVo/CaCo-2 (colon carcinoma), MCF-7 (breast adenocarcinoma), and T98G (glioma) cells [158,161,164].

Zinc oxide NPs have also been studied as tools for the targeted delivery of chemotherapeutics [158,161,162,163,164]. Photo-stimulated, paclitaxel- and cisplatin-loaded ZnO QDs were also used as theranostics against HNSCC cells under UV-A irradiation [161]. Peng et al. [162] reported that VP-16 (etoposide) was loaded into beta-cyclodextrin functionalized iron oxide QDs, coated with ZnO and doped with Er^3+^ and Yb^3+^ (Fe_3_O_4_@ZnO:Er^3+^,Yb^3+^@β-CD). After microwave-triggering, VP-16 released from Fe_3_O_4_@ZnO:Er^3+^,Yb^3+^@β-CD NPs demonstrated antitumor activity in MCF-7 cells. Doxorubicin [163] and daunorubicin [28,164] were delivered to MCF-7, A549, K562 (sensitive leukemia), and K562/A02 (resistant leukemia) cells, using ZnO NP-mediated drug delivery systems. Doxorubicin was loaded onto ZnO NPs at a concentration up to 0.1 mg/mL and stabilized with starch [163]. The doxorubicin-loaded ZnO NPs showed antiproliferative activity against MCF-7 cells. Daunorubicin was incorporated into multilamellar liposomes with hexagonal ZnO NP cores for pH-sensitive drug release against cancer cells [164]. It was also delivered by ZnO NPs capped with aminopolysiloxane to generate synergistic anticancer activity against leukemia cells [28,164].

### 5.2. Antidiabetic Activity

Zinc can ameliorate type 1 and type 2 diabetes because of its role in the function of enzymes (>300) needed to maintain metabolic homeostasis in the body [165,166]. As an essential micronutrient, zinc is involved with the synthesis, storage, and secretion of insulin [167]. Specifically, zinc enhances the structural integrity of insulin through zinc–insulin hexamers. Zinc also downregulates blood glucose levels by inhibiting glucose absorption and increasing glucose uptake by skeletal muscle and adipose tissue. El-Gharbawy et al. [165] reported that hexagonal ZnO NPs and vildagliptin (10 mg/kg/day, oral administration), an antidiabetic drug, restored the structure and function of beta cells in a model of type 2 diabetes. They used sol–gel synthesis to produce mixed shapes of oval- and rod-shaped ZnO NPs (~20 nm). Umrani and Paknikar [166] also described the antidiabetic activity of hexagonal ZnO NPs (10–15 nm) in rat models of type 1 and 2 diabetes.

### 5.3. Antimicrobial Activity

**Figure 3:**
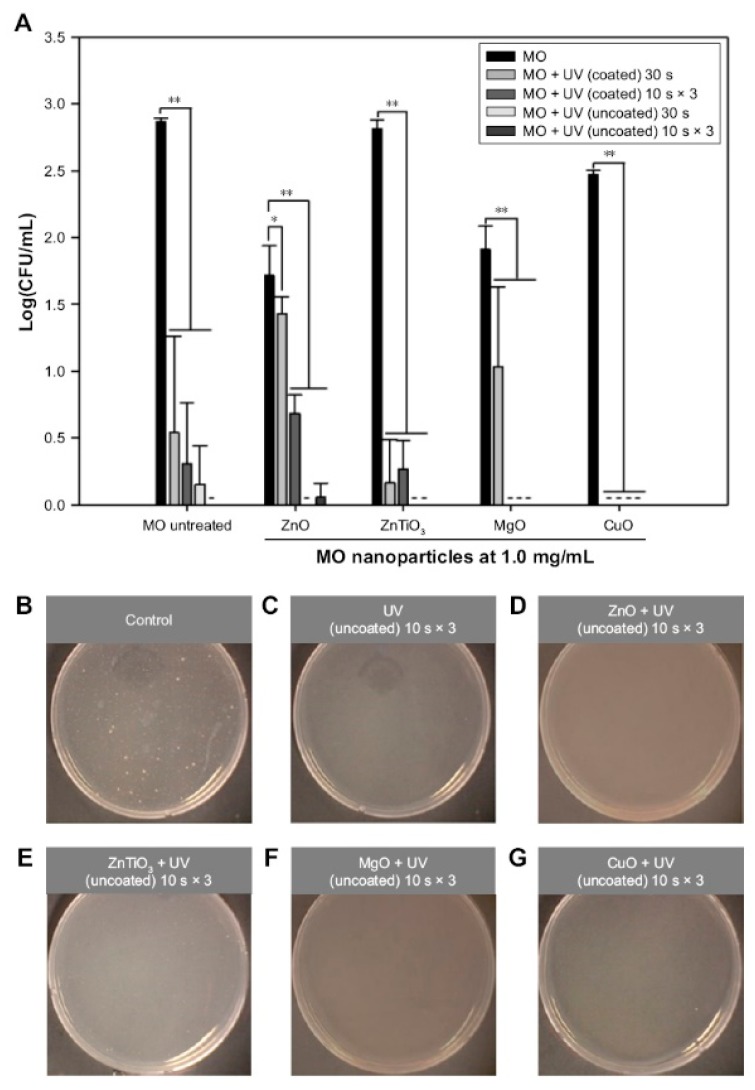
Antibacterial effects of dual UV-MO NPs hybrid on *Escherichia coli*. (**A**) The plot of Log(CFU/mL) versus MO NPs. Dual UV was exposed for 30 s or 10 s in three cycles, while MO NPs at 1.0 mg/mL were treated for 30 min. Representative plate images of colonies after the treatment of cyclic exposure (10 s × 3) from the uncoated area and MO NPs are shown: (**B**) control (untreated), (**C**) UV (uncoated), (**D**) ZnO, (**E**) ZnTiO_3_, (**F**) MgO, and (**G**) CuO, -, not detected; * *p* < 0.05; ** *p* < 0.01. Reproduced with permission from Jin et al., International Journal of Nanomedicine; published by DOVE Medical Press, 2017. from ref [116]. Abbreviations: UV, ultraviolet; MO, metal oxide; NPs, nanoparticles; ZnO, zinc oxide; ZnTiO_3_, zinc titanate; MgO, magnesium oxide; CuO, cupric oxide.

Zinc oxide NPs produce antimicrobial activity via adsorption-induced membrane damage and ROS-mediated cellular toxicity [6,116,158]. They are effective against *Escherichia coli*, *Staphylococcus aureus*, *Pseudomonas aeruginosa*, *Pseudomonas vulgaris*, *Bacillus subtilis*, *Bacillus megaterium*, *Sarcina lutea*, *Klebsiella pneumonia*, *Candida albicans*, and *Aspergillus niger* [6,13,34]. Zinc oxide NPs were used to deliver gentamicin from the intra- and interparticle pores of host–guest structures [6,121]. Jin et al. [13] presented the antibacterial activity of a self-assembled ZnO NP network structure with macro- and mesopores on a Si wafer against *E. coli* under dual UV irradiation. Jin et al. [116] also demonstrated the antibacterial and antiviral activities of hexagonal ZnO NPs (<100 nm in diameter) with and without UV-A and UV-C irradiation. The antimicrobial activity of ZnO NPs at a concentration of 1.0 mg/mL was tested in *E. coli* (Figure 3) and M13 bacteriophages.

### 5.4. Anti-Inflammatory Activity

Zinc oxide NPs have anti-inflammatory activity in response to pathogens or chemicals [168]. Zinc oxide NPs reduce inflammation by (i) blocking the production of pro-inflammatory cytokines such as interleukin (IL)-1β and IL-18 via inhibiting NF-kB and caspase 1 in activated mast cells and macrophages; (ii) inhibiting mast cell proliferation by increasing p53 and decreasing thymic stromal lymphopoietin production related to IL-13, a T_H_2 cytokine, along with IL-1 and tumor necrosis factor-α; and (iii) suppressing lipopolysaccharide-induced cyclooxygenase-2 and inducible nitric oxide synthase expression [168,169]. Wiegand et al. [10] described a ZnO-functionalized textile (Benevit Zink+, Benevit Van Clewe, Dingden, Germany) made of 74% Lyocell fiber, 19% SmartCell sensitive fiber, and 7% spandex. This ZnO-functionalized textile increased the antioxidative capacity and reduced bacterial growth on the skin of atopic dermatitis patients. Yao et al. [170] also reported that ZnO NP-embedded titanium dioxide (TiO_2_) nanotubes had antibacterial and anti-inflammatory activities. A multifunctional microstructure containing ZnO NPs trapped ibuprofen, an anti-inflammatory drug, in the intra- and interparticle pores of a magnesium/epoxy resin-ZnO/polycaprolactone [171].

### 5.5. Wound Healing

**Figure 4:**
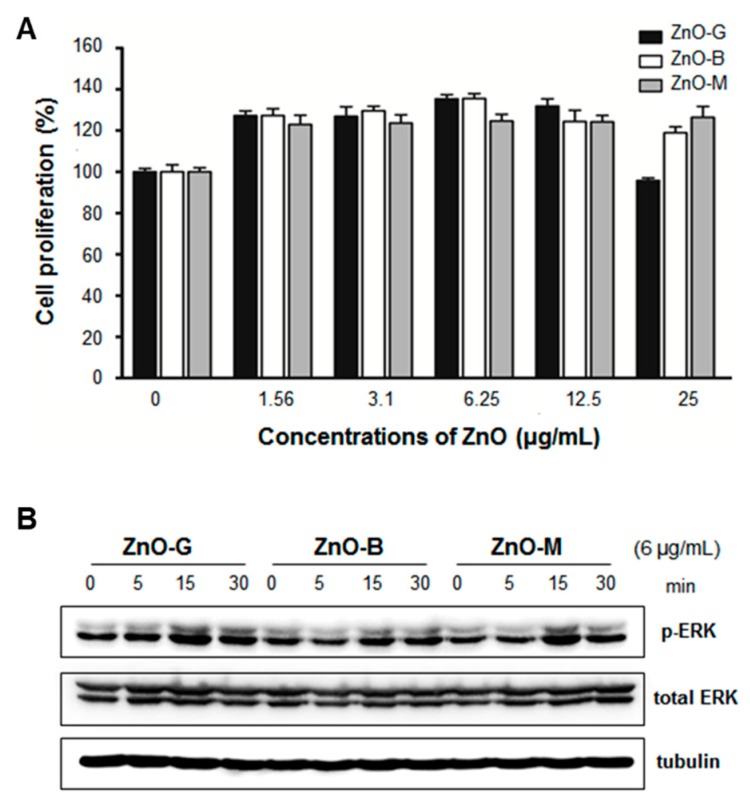
ZnO nanorod enhancement of ADSC proliferation based on the activation of ERK. (**A**) Proliferation (%) of ADSCs using ZnO nanorods at concentrations ranging from 1.56 to 25 μg/mL and (**B**) ERK protein expression with and without phosphorylation determined using the western blotting method. * *p* < 0.05; ** *p* < 0.01. Reproduced with permission from Jin et al., Tissue Engineering Part C: Methods; published by Mary Ann Liebert, Inc., 2016. from ref [178]. Abbreviations: ZnO, zinc oxide; ADSC, adipose-derived stem cell; ERK, extracellular-signal-regulated kinase.

**Figure 5:**
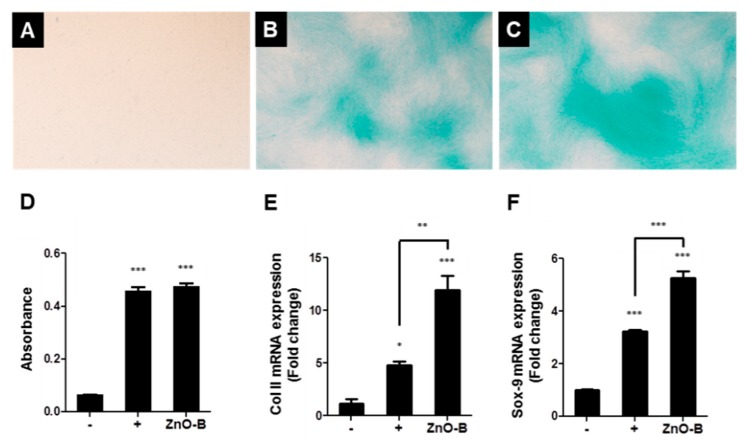
The effect of ZnO nanorods on chondrogenic differentiation in ADSCs. (**A**) negative control (basal medium, expressed as “-” at (**D**–**F**)), (**B**) positive control (chondrogenic medium and 5% Dex, expressed as “+” at (**D**–**F**)), (**C**) ZnO-B treatment at 6 μg/mL, and (**D**) absorbance of Alcian blue extracts at 610 nm and qPCR data of (**E**) Col II and (**F**) Sox-9 mRNA expression after 21 days with or without ZnO-B. Images were taken at × 40 magnification. * *p* < 0.05; ** *p* < 0.001; *** *p* < 0.0001. Reproduced with permission from Jin et al., Tissue Engineering Part C: Methods; published by Mary Ann Liebert, Inc., 2016. from ref [178]. Abbreviations: ZnO, zinc oxide; ADSC, adipose-derived stem cell; Dex, dextrose.

As an essential micronutrient, zinc plays the following key roles in wound repair: it contributes to (i) fibrin clot formation, (ii) resolution of the inflammatory response, (iii) induction of cell proliferation, re-epithelization, granulation, and angiogenesis, and (iv) remodeling of the extracellular matrix [172,173]. By providing a prolonged supply of zinc to wounds, ZnO NPs are attractive emerging therapeutic agents to effectively penetrate the cell, to modulate the immune system, and to promote disinfection. Their promoted antibacterial action and enhanced re-epithelization have also been reported in several studies of wounds [174,175]. Gao et al. [176] explored using ZnO NPs as antimicrobial tissue adhesives for the closure of skin wounds. Alginate/nano-ZnO composite bandages have also been used on wounds that were infected with *S. aureus* and *E. coli* [177]. Jin et al. [178] reported that ZnO nanorods enhanced the proliferation of adipocyte-derived stem cells (ADSCs) for tissue engineering. The nanorods were synthesized by grinding (ZnO-G), boiling (ZnO-B), and micelle formation (ZnO-M) techniques. Zinc oxide nanorods (6 μg/mL) enhanced ADSC proliferation via increased phosphorylation of extracellular-signal-regulated kinase (ERK) (Figure 4). Based on proteomic approaches using tandem mass spectrometry (MS/MS), thioredoxin (Trx) was related to the enhanced rate of ADSC proliferation seen after treatment with the ZnO nanorods. The ZnO nanorods increased the expression of thioredoxin reductases (TrxR) I mRNA, while the increase in the proliferation rate was abolished following treatment with epigallocatechin gallate (EGCG) (10 mM), a TrxR I blocker. They had chondrogenic differentiation capacity in ADSC, which Col II and Sox-9 mRNA expressions increased (Figure 5). Topical pharmaceutical formulations of Zn/(Cu) and ZnO/cod liver oil regulated the rates of cellular proliferation and reepithelization [179,180]. In particular, the ZnO/cod liver oil ointment efficiently increased the rate of wound healing after it was delayed by dexamethasone treatment [180].

### 5.6. Imaging Agents

Quantum dots are semiconductors of transparent nanoparticles (1–10 nm) [181]. They have unique optical and electronic properties, including fluorescence under light sources for bioimaging applications [182]. In core–shell configurations, the photoluminescent quantum yield of the core emission is boosted and shielded from photobleaching [181,182]. In pharmaceutical and biomedical applications, QDs can be used for imaging and drug delivery [181,182]. Liu et al. [183] described the use of folic acid-modified ZnO nanocrystals for near-infrared excitation. Jia and Misra [184] also described photoluminescent ZnO QDs (3–4 nm) that were immobilized on SiO_2_ nanospheres (~150–200 nm) for bioimaging purposes.

### 5.7. Sensors

Zinc oxide NPs have been used as biomedical diagnostic/analytical sensors for detecting gases and biochemicals [57,185,186]. In gas sensors, the pore properties are important factors because they allow adsorbates into internal surfaces to ensure adequate adsorption performance. For example, highly sensitive and selective gas sensors of ZnO nanowires/NPs were able to detect ethanol and acetone quickly and accurately [187,188]. Nano-brush and pearl chain-like ZnO nanowires were developed for the selective and sensitive detection of ethanol [187]. Zhou et al. [188] reported an interlocking *p* + *n* field-effect transistor circuit of Mn-doped ZnO NPs for detecting acetone as low as 2 ppm, even under conditions of high relative humidity (>85%). Zinc oxide nanorod field-effect transistors (FETs) were monitored physiological conditions via the detection of glucose, cholesterol, and urea in the samples of mice’s blood, and diabetic dogs’ serum and blood [57]. Mohsin et al. [189] reported using aligned ZnO nanorods for epinephrine sensing. Zinc oxide electrodes on flexible porous polyimide substrates were also developed for the detection of cardiac troponin [190]. Perumal et al. [191] reported gold (Au)–ZnO hybrid NP films for optical and impedimetric analyses.

## 6. Conclusions

Zinc oxide NPs have different physicochemical characteristics that can vary depending on the techniques used for synthesis. The different physicochemical properties of ZnO NPs affect their biomedical activity in vitro and in vivo. Synthetic ZnO NPs form one-, two-, and three-dimensional structures or hierarchically porous network structures that enhance their performance. They show promising potential as therapeutics with anticancer, antidiabetic, antimicrobial, anti-inflammatory, and wound healing activities. Zinc oxide NPs are also used for imaging tools and biosensors. In the near future, it is expected that ZnO NPs can be extensively applied in non-clinical and clinical studies as emerging therapeutic agents.

## Figures and Tables

**Table 1:** Current applications of ZnO NPs.

Category	Applications	References
Pharmaceuticals	▪ Soap ▪ Ointment ▪ Dental inlays ▪ Food powders	[20]
Cosmetics—hair and skin care products	▪ Powders ▪ Creams ▪ UV radiation-blocking sunscreen lotions ▪ Burn ointments	[20,21]
Medical devices	▪ Surgical/industrial adhesives ▪ Mastics ▪ Sealants	[22]

**Table 2:** Techniques for the synthesis of ZnO NPs.

Synthesis Technique		Advantages	Disadvantages	References
Physical methods	- Arc plasma - Thermal evaporation - Physical vapor deposition - Ultrasonic irradiation - Laser ablation	▪ Simple ▪ Low cost ▪ Catalyst-free ▪ Industrial-scale production	▪ Parameter control	[9,54,55,56,57,58,59,60,61,62,63,64,65,66,67]
Chemical methods	- Microemulsion - Sol–gel - Precipitation - Hydrothermal method - Solvothermal method - Chemical vapor deposition	▪ Inexpensive and easy-to-handle chemical reagents ▪ Uncomplicated equipment ▪ Low energy input ▪ Easy parameter tailoring ▪ Industrial-scale production	▪ Surfactant use ▪ High cost of precursors	[9,35,55,68,69,70,71,72,73,74,75,76]
Biological methods (green synthesis)	- Plant extracts - Microorganisms - Biotechnology method - Biochemistry method	▪ Promising alternatives to chemical methods ▪ Eco-friendly ▪ Non-toxic (safer) ▪ Inexpensive organic solvents	▪ Nanoparticle stability ▪ Antimicrobial activity ▪ Unclear mechanism	[9,52,56,77,78,79,80,81,82,83,84,85,86,87,88,89,90,91,92,93,94,95,96,97]
Microfluidic reactor-based methods	- Continuous flow - Segmented flow - Co-flow	▪ High value-added products ▪ Reproducible ▪ Non-toxic	▪ Parameter control	[98,99,100,101,102,103,104,105,106,107]

**Table 3:** Techniques for analyzing the physicochemical properties of ZnO NPs.

Physicochemical Characteristics	Analysis Techniques
Agglomeration/aggregation	SEM (++), TEM (++), SPM (++), MALS (+), SAXS (+/−), SMPS (++)
Composition	Neutron/electron scattering (+), XRD (+), ICP-MS/OES (++), SP ICP-MS (++), EDS (+), NMR (++), XRF (++), SIMS (+), EELS (+), TOF-MS/ATOF-MS (++), FTIR/RS (++), UV–Vis (+), AES (+/−)
Crystalline phase	SEM (+), TEM (+), Neutron/electron scattering (++), XRD (++), FTIR/RS (+; RS), TGA/DSC (+)
Dustiness	SD/VS (+)
Solubility	DLS/PCS/QELS (++), MALS (++)
Dispersibility	DLS/PCS/QELS (++), MALS (++)
Stability	DLS/PCS/QELS (++), MALS (++), ELS (++), TGA/DSC (++)
Particle size/size distribution	SEM (++), TEM (++), SPM (++), DLS/PCS/QELS (++), MALS (++), SAXS (+), XRD (+; volume weighted primary crystals), SP ICP-MS (++), TOF-MS/ATOF-MS (+; coupled with FFF), FTIR/RS (+; RS), UV–Vis (+; for plasmonic materials), CHDF (++), FFF/A4F/FlFFF (++), BET (+/−), CLS (++), SMPS (++)
Shape	SEM (++), TEM (++), SPM (++)
Specific surface area	TEM (+; electron tomography), SAXS (+/−), BET (++)
Surface chemistry	ICP-MS/OES (+/−), EDS (+), NMR (+), XPS (++), SIMS (++), EELS (++), TOF-MS/ATOF-MS (++), FTIR/RS (+), AES (++), TGA/DSC (++)
Surface charge/zeta potential	SPM (+/−), DLS/PCS/QELS (+), ELS (++)
Porosity	BET (++), Mercury intrusion (++)

Abbreviations: SEM: scanning electron microscopy, TEM: transmission electron microscopy, SPM: scanning probe microscopy, MALS: multiangle light scattering, SAXS: small-angle X-ray scattering, SMPS: scanning mobility particle sizer, XRD: X-ray diffraction, ICP-MS: inductively coupled plasma-mass spectroscopy, OES: optical emission spectrometer, SP ICP-MS: single particle ICP-MS, EDS: energy dispersive X-ray spectroscopy, NMR: nuclear magnetic resonance, XRF: X-ray fluorescence spectrometer, SIMS: secondary ion mass spectrometry, EELS: electron energy loss spectroscopy, TOF-MS: time-of-flight mass spectrometry, ATOF-MS: aerosol TOF-MS, FTIR: Fourier-transform infrared spectroscopy, RS: Raman spectroscopy, UV–Vis: ultraviolet–visible spectroscopy, AES: Auger electron spectroscopy, TGA: thermogravimetric analysis, DSC: differential scanning calorimetry, SD: small drum rotator, VS: vortex shaker, DLS: dynamic light scattering, PCS: photon correlation spectroscopy, QELS: quasi-elastic light scattering, ELS: electrophoretic light scattering, CHDF: capillary hydrodynamic flow fractionation, FFF: field flow fractionation, A4F: asymmetrical flow field-flow fractionation, FlFFF: flow field-flow fractionation, BET: Brunauer–Emmett-Teller analysis, CLS: centrifugal liquid sedimentation.

**Table 4:** Biomedical applications of ZnO NPs.

Biomedical Application	Morphology/Structure	Test System	References
**Anticancer activity**			[1,2,5]
Paclitaxel or cisplatin-ZnO	Photo-stimulated paclitaxel or cisplatin-ZnO NPs under UV-A irradiation	HNSCC cells	[161]
VP-16-Fe_3_O_4_@ZnO:Er^3+^,Yb^3+^@β-CD	VP-16 released from Fe_3_O_4_@ZnO:Er^3+^,Yb^3+^@β-CD NPs after microwave-triggering	MCF-7 cells	[162]
Doxorubicin-ZnO	Starch-stabilized ZnO NPs	MCF-7 cells	[163]
Daunorubicin-ZnO	Multilamellar liposomes with hexagonal ZnO NP cores	A549 (non-small cell lung carcinoma) cells	[164]
	Aminopolysiloxane-capped ZnO NPs	K562 (sensitive leukemia) and K562/A02 (resistant leukemia) cells	[28]
**Antidiabetic activity**			[165,166,167]
Vildagliptin + ZnO	Hexagonal ZnO NPs (mixed shape,~20 nm)	Rats, type 2 diabetes	[165]
ZnO	Hexagonal ZnO NPs (spherical shape, 10–15 nm)	Rats, type 1 and 2 diabetes	[166]
**Antimicrobial activity**			[6,34]
ZnO	Self-assembled ZnO NP network structure on Si wafer under dual UV irradiation (ZnO 0.05 mg/mL, UV 10 sec, 5 or 120 min incubation)	*E. coli*	[13]
ZnO	Hexagonal ZnO NPs with/without dual UV irradiation (~100 nm, ZnO 1.0 mg/mL, UV 30 sec, 30 min incubation)	*Escherichia coli*, M13 bacteriophages	[116]
Gentamicin + ZnO	Mesoporous ZnO structures on Si substrates (guest-host structures)	In vitro release for 7 days	[121]
**Anti-inflammatory activity**			[168,169]
ZnO (74% Lyocell fiber, 19% Smart Cell sensitive fiber, and 7% spandex)	ZnO-functionalized textile (Benevit Zink+)	*Staphylococcus aureus*, *Klebsiella pneumoniae* (for atopic dermatitis patients)	[10]
ZnO–TiO_2_	ZnO NP-embedded TiO_2_ nanotubes	Macrophage-like RAW 264.7 (murine leukemic monocyte) cells, *S. aureus*	[170]
Magnesium/epoxy resin-ZnO/poly-capro- lactone-ibuprofen	Multifunctional microstructure (coating)	In vitro release	[171]
**Wound healing**			[172,173,174,175]
ZnO	ZnO NPs (antimicrobial tissue adhesive, 71.1 nm)	Skin wound closure (*E. coli* and adhesion test)	[176]
Alginate/ZnO	Alginate/nano-ZnO composite bandages	Infected wounds (*S. aureus* and *E. coli*)	[177]
ZnO	ZnO NPs (boiling method-based synthesis)	Wound dressing (adipocyte-derived stem cell proliferation)	[178]
ZnO	Topical ZnO formulations (Increased local Zn and basal cell metallothionein in wound margins for accelerated wound healing)	Wound dressing (surgical wound model in Sprague-Dawley rat)	[179]
Cod liver oil/ZnO	Zincojecol (ointment containing cod liver oil and ZnO)	Wound dressing (tail skin, retarded wound model by dexamethasone)	[180]
**Imaging agents**			[181,182]
Folic acid-ZnO QD	Folic acid-modified ZnO nanocrystals (NIR excitation)	KB (oral carcinoma) cells	[183]
ZnO QD	ZnO QDs (3–4 nm) immobilized on silica nanospheres (~150–200 nm) (photoluminescence)	Photoluminescence intensity	[184]
**Sensors**			[185,186]
ZnO	Three-dimensional interconnected ZnO nanostructures (macro-mesoporosity)	Acetone/methanol detection	[29]
ZnO	ZnO nano-brush and pearl chain-like nanowire	Selective/sensitive ethanol sensing	[187]
Mn-ZnO	Interlocking *p* + *n* field-effect transistor circuit of Mn-doped ZnO NPs	Acetone sensing (> 2 ppm)	[188]
ZnO	Aligned ZnO nanorods	Epinephrine sensing	[189]
ZnO	ZnO electrodes on flexible porous polyimide substrates	Cardiac troponin sensing	[190]
ZnO	ZnO nanorod field-effect transistors (FETs)	Glucose, cholesterol, and urea sensing	[57]
Au–ZnO	Gold (Au)–ZnO hybrid NP films	Optical and impedimetric analyses	[191]
